# Comparison of hand hygiene antimicrobial efficacy: Melaleucaalternifolia essential oil versus triclosan versus chlorhexidine

**DOI:** 10.1186/2047-2994-4-S1-P306

**Published:** 2015-06-16

**Authors:** JR Gnatta, FMG Pinto, CQM Bruna, MC Padoveze, KU Graziano, MJPD Silva

**Affiliations:** 1 University Hospital, University of Sao Paulo, Sao Paulo, Brazil; 2 School of Nursing, University of Sao Paulo, Sao Paulo, Brazil

## Introduction

The antimicrobial effects of essential oils have been reported in the scientific literature, especially regarding the essential oil of *Melaleucaalternifolia*, also known as tea tree essential oil (TTO). This essential oil has antiseptic properties and can represent a natural-product alternative for hand hygiene in health-care settings which currently use mainly products based on triclosan and chlorhexidine.

## Objectives

To evaluate the antimicrobial efficacy in hand hygiene performed using three distinct soaps containing 2.0% *Melaleucaalternifolia* essential oil; 0.5% triclosan; 2.0% chlorhexidine.

## Methods

Was applied the methodology of the European Committee for Standardization, EN1499 version April 2013, indicated to evaluate the efficacy of antiseptics for hand hygiene. The hands of 15 healthy volunteers were artificially contaminated with *Escherichia coli* K12 and then the hands were washed with each of the products being assessed or the reference soap (soft soap). The number of microorganisms was counted before (pre-values) and after (post-values) each procedure and microbial logarithmic reduction was performed for each of the participants in each procedure. Data were analyzed using two non-parametric tests: Wilcoxon test and Friedman test. Level of significance p=0.01 one-sided.

## Results

When the Wilcoxon test was applied, the three test products showed to be superior to soft soap (chlorhexidine, p=0.003, triclosan and TTO p<0.001), whereas the soaps containing triclosan or TTO were superior in efficacy to soap containing chlorhexidine. When Friedman’s test was applied, products that showed superior efficacy when compared to the soft soap were those containing 0.5% triclosan (p<0.001) and 2.0% TTO (p<0.001) and when efficacy was compared between the products, the three were equivalent, with no superiority between them.

## Conclusion

When the Wilcoxon test is used, the three soaps can be considered antimicrobial, with the soaps containing 2.0% TTO and 0.5% triclosan being superior to the soap with 2.0% CHX. If Friedman’s test is used, only the 0.5% triclosan soap and the soap containing 2.0% TTO can be considered antimicrobial and both showed similar antimicrobial efficacy.

## Disclosure of interest

None declared.

